# A Post Hoc Analysis of Efficacy Data on Sarecycline in Hispanics with Acne from Two Phase 3, Multicenter, Randomized, Double-Blind, Placebo-Controlled Clinical Trials

**DOI:** 10.3390/antibiotics12010089

**Published:** 2023-01-04

**Authors:** Angela Yen Moore, Kara Hurley, Stephen Andrew Moore

**Affiliations:** 1 Arlington Center for Dermatology, Arlington, TX 76011, USA; 2 Arlington Research Center, Arlington, TX 76011, USA; 3 Department of Dermatology, Baylor University Medical Center, Dallas, TX 75246, USA; 4 Department of Medical Education, Texas Christian University School of Medicine, Fort Worth, TX 76107, USA; 5 Texas College of Osteopathic Medicine, University of North Texas Health Science Center, Fort Worth, TX 76107, USA

**Keywords:** acne vulgaris, skin color, tetracycline, sarecycline, Hispanic

## Abstract

Sarecycline is a novel, narrow-spectrum, third generation tetracycline class antibiotic approved by the Food and Drug Administration (FDA) for the treatment of moderate-to-severe acne in patients ages nine and older. Recently, focus has increased on whether treatment responses differ in acne in skin of color. Here, we aimed to analyze the efficacy of using sarecycline in Hispanics. We report pooled post hoc analysis of efficacy data on sarecycline in Hispanics with acne from two phase 3, multicenter, randomized, double-blind, placebo-controlled clinical trials, SC1401 and SC1402. Of 2002 patients in the pooled trials with moderate-to-severe acne, 26.9% were Hispanic. Facial inflammatory lesion counts decreased as early as week 3 by 26% (*p* = 0.0279), with continued reduction by 41% by week 6 (*p* = 0.0003), by 51% by week 9 (*p* < 0.0001), and by 55% by week 12 (*p* < 0.0001). Acne is the most common skin condition diagnosed in Hispanics, and this study illustrates a statistically significant reduction in acne in Hispanic patients with moderate-to-severe acne treated with oral sarecycline. Therefore, oral sarecycline shows promising results as a safe and effective treatment for acne in Hispanics.

## 1. Introduction

Comprising 18% of the U.S. population, the Hispanic population rose to 60.6 million in 2019 [1]. As the most common dermatologic diagnosis in Hispanics, acne affects more Hispanic (32%) than Caucasian (24%) women [2]. A population-based, self-reported, cross-sectional survey of 2895 women ages 10 to 70 found the prevalence of acne to be greater in African-Americans and Hispanics than in Caucasians, Asians, and continental Indians with the conclusion that acne was more prevalent in darker skin types [3]. Focus on acne across different races and ethnicities is important as skin color can sometimes guide treatment recommendations [4].

Recent guidelines of care for the management of acne vulgaris have been published [5]. Current treatments include topical and systemic agents. First line treatment for mild acne consists of benzoyl peroxide, topical retinoids, and/or topical antibiotics. Moderate to severe acne is treated with an oral antibiotic in addition to the topical therapies mentioned prior. Oral isotretinoin can be used to treat severe acne, but carries significant adverse effects [5]. Sarecycline is a narrow-spectrum, third generation tetracycline class antibiotic, approved by the Food and Drug Administration (FDA) for the treatment of moderate-to-severe acne in patients ages nine and older [6,7]. Tetracycline mechanism of action includes inhibition of protein synthesis through interaction with the 30S bacterial ribosome [7]. Sarecycline differs from other tetracyclines in a 7-[methoxy(methyl)amino]methyl] group attached at the C7 position. This C7 extension is significant as it was found to extend into the messenger RNA (mRNA) channel and have direct interaction with the A-site codon, possibly interfering with mRNA movement and/or inhibiting A-site codon–anticodon interaction. This interaction is believed to contribute to increased inhibition of sarecycline [7].

With increasing focus on whether responses differ in acne in skin of color, we report pooled post hoc analysis of efficacy data on sarecycline in Hispanics with acne from two Phase 3, multicenter, randomized, double-blind, placebo-controlled clinical trials [8]. Therefore, this study included 2002 patients in the United States with moderate-to-severe acne and aimed to analyze the efficacy of using sarecycline in Hispanics.

## 2. Results

### 2.1. Baseline Lesion Count and Severity

Of 2002 patients with moderate-to-severe acne, 550 (27.5%) were Hispanic. Of the Hispanic patients, 60% were females and 40% were males At baseline, the mean facial inflammatory lesion count was 29.70 vs. 30.22, and the mean facial non-inflammatory lesion count was 42.23 vs. 44.77 (sarecycline vs. placebo, respectively). Of the total intent-to-treat (ITT) Hispanic population in the study (n = 550), 79.26% had moderate acne, with Investigator Global Assessment IGA of 3, and 20.74% had severe acne, with IGA of 4.

### 2.2. Clinical Outcomes

**Figure 1:**
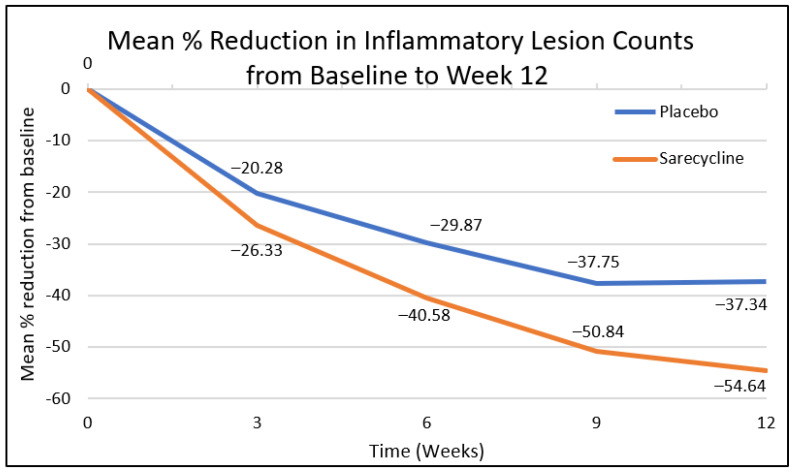
Mean % reduction of facial inflammatory lesion count from baseline to week 12.

For the facial inflammatory lesion count, statistically significant reduction was observed at week 3 by 26% vs. 20%, (confidence interval (CI): −6.06 (−11.45, −0.66), *p* = 0.0279), at week 6 by 41% vs. 30% (CI: −10.70 (−16.48, −4.93), *p* = 0.0003), at week 9 by 51% vs. 38% (CI: −13.09 (−19.18, −6.99), *p* < 0.0001), and at week 12 by 55% vs. 37% (CI: −17.29 (−24.39, −10.20), *p* < 0.0001) in patients on sarecycline vs. placebo, respectively (Figure 1).

Non-inflammatory (comedonal) facial lesions in Hispanics decreased by 19% vs. 16% at week 3 (confidence interval (CI): −3.60 (−9.14, 1.95), *p* = 0.2031), 31% vs. 25% at week 6 (CI: −6.20 (−13.24, 0.84), *p* = 0.0842), and a statistically significant reduction by 41% vs. 28% at week 9 (CI: −6.20 (−13.24, 0.84), *p* = 0.0006) and by 41% vs. 28% at week 12 (CI: −12.91 (−20.69, −5.13), *p* = 0.0012) in patients on sarecycline vs. placebo, respectively.

### 2.3. Coprimary Efficacy Endpoints

**Figure 2:**
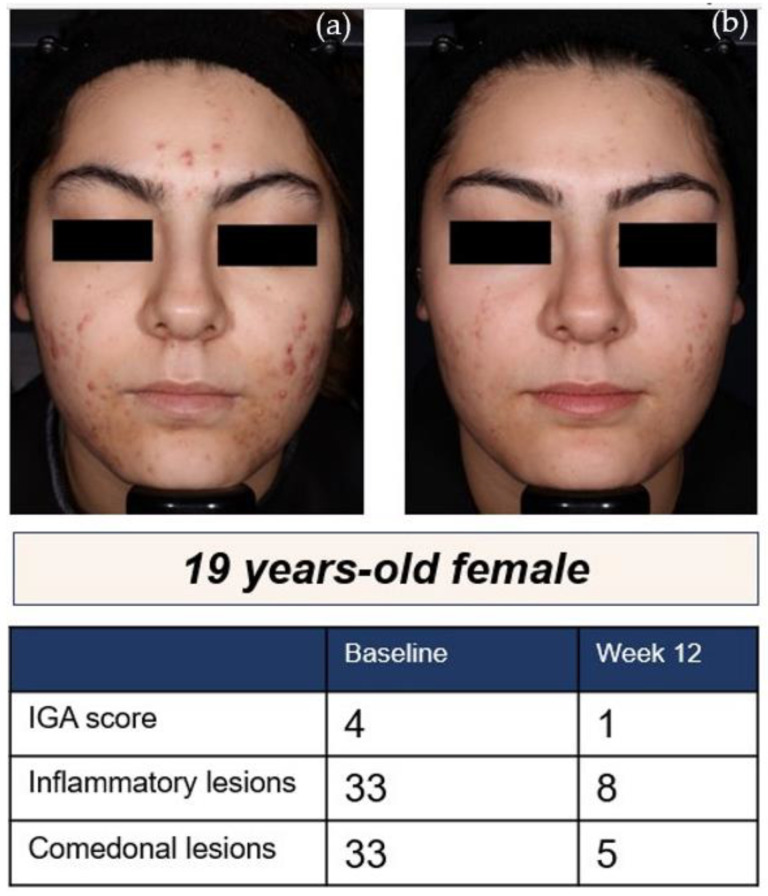
A 19-year-old Hispanic female [8]: (**a**) at baseline and; (**b**) after 12 weeks of treatment with sarecycline 1.5 mg/kg/day.

At week 12, coprimary efficacy analyses revealed statistically significant change from baseline (CFB) in inflammatory lesion counts with sarecycline vs. placebo, respectively (CI: −5.19 (−7.23, −3.14), *p* < 0.0001), and a statistically significant IGA success (defined as two severity grade reduction and clear or almost clear) was achieved in 24% vs. 15% of Hispanics on sarecycline vs. placebo, respectively (CI: 10.13 (3.65, 16.62), *p* = 0.0023) (Figure 2).

## 3. Materials and Methods

### 3.1. Ethics

SC1401 and SC1402 were conducted in accordance with good clinical practice (GCP). The protocol and supporting documents were reviewed and approved by an appropriately constituted Institutional Review Board (IRB). Written informed consent (and/or assent) was obtained from each subject.

### 3.2. Study Design

This is a post hoc analysis of phase 3 clinical trials SC1401 and SC1402. Patients were randomized 1:1 to receive once-daily oral sarecycline at 1.5 mg/kg/day or placebo. The patients were assessed by a dermatologist at baseline, 3, 6, 9, and 12 weeks. Patients were assigned a subject study identification number using the Interactive Response Technology (IRT) system. The IRT system was used for randomization, assigning the subject to a double-blind treatment group. The coprimary efficacy endpoints of the study were the absolute change from baseline (CFB) in the facial inflammatory lesion count at week 12 and a dichotomized IGA at week 12 (dichotomized to reflect either ‘success’ or ‘failure’ with ‘success’ defined as at least a 2-point decrease from baseline in the IGA assessment and a score of clear [0] or almost clear [1] on the IGA assessment).

### 3.3. Patient Characteristics

Inclusion criteria were defined as, ages 9 to 45, has a body weight between 33 kg and 136 kg, has facial acne vulgaris with 20 to 50 inflammatory lesions (papules, pustules, and nodules), up to 100 noninflammatory lesions (open and closed comedones), no more than two nodules, and an IGA score of moderate (3) or severe (4). Exclusion criteria consisted of dermatologic conditions of the face (i.e., sunburn, skin burn, drug-induced acne), facial hair, alllergy to tetracycline-class antibiotics, a history of pseudomembranous colitis or antibiotic-associated colitis, and use of systemic retinoids, systemic corticosteroids, androgens/anti-androgenic therapy, and/or non-medicated procedures for the treatment of acne within 12 weeks of randomization.

### 3.4. Methods

In SC1401 and SC1402, the absolute change from baseline in inflammatory lesion counts was analyzed using an analysis of covariance (ANCOVA) model with factors for treatment and (pooled) site and the baseline value as a covariate. The adjusted means with associated 95% confidence intervals (CIs) from the ANCOVA model was presented for each treatment and for the difference between the treatments. IGA success was analyzed using a Cochran–Mantel–Haenszel (CMH) test with adjustment for (pooled) site. The same methods were applied to the post hoc analysis.

## 4. Discussion

Studies have demonstrated that acne is highly prevalent in Hispanic women [2,3], but data on male prevalence are lacking. In 550 Hispanic subjects in phase 3 clinical trials SC1401 and SC1402, facial inflammatory lesion counts decreased as early as week 3 by 26% (*p* = 0.0279), with continued reduction by 41% by week 6 (*p* = 0.0003), by 51% by week 9 (*p* < 0.0001), and by 55% by week 12 (*p* < 0.0001). Sarecycline was approved by the FDA in 2018 for the treatment of moderate to severe acne. Sarecycline is a narrow-spectrum tetracycline with potent activity against Gram-positive bacteria in vitro, including *Cutibacterium acnes*, a bacteria implicated in the pathogenesis of acne. Sarecycline also has anti-inflammatory properties consistent with other tetracyclines, in which the mechanism is not fully understood [6,7]. In a systematic review of sarecycline of phase 2 and phase 3 trials, the most common adverse effects were nausea in 2.2% vs. 1.2%, vomiting in 1.0% vs. 0.7%, nasopharyngitis in 2.8% vs. 2.3%, and headaches in 2.8% vs. 3.8% of sarecycline vs. placebo subjects, respectively [9]. Less than 1.1% of subjects displayed vestibular, phototoxic, vulvovaginal candidiasis, and mycotic infections. No symptoms of special interest were reported for diarrhea, esophagitis, pseudotumor cerebri, blurry or double vision, dizziness, vertigo, and blue-gray pigmentation [9].

Tetracyclines exhibit bacteriostatic effects by binding to the 30S subunit of the bacterial ribosome. Sarecycline possesses a unique C7 extension that directly interacts with the mRNA channel. To our knowledge, no studies exist directly comparing the efficacy of sarecycline to that of other tetracyclines. Doxycycline is currently the first-line systemic antibiotic for acne [5]. Minocycline is another tetracycline commonly used to treat acne. A Cochrane review of clinical trials found minocycline to be effective but not superior to other antibiotics in the treatment of acne. Other antibiotics such as macrolides have been studied in acne. Macrolides are bacteriostatic by inhibiting the 50S subunit of the bacteria ribosome. Azithromycin is the most well studied macrolide for acne therapy. A recent meta-analysis of randomized controlled trials that compared the efficacy of azithromycin pulse therapy with that of daily doxycycline showed that azithromycin is equivalent to doxycycline at 12 weeks in the treatment of severe acne [10]. No standard pulse dosing regimen has been established. Reported studies range from 3 times a week to 4 days a month [5]. Trimethoprim-sulfamethoxazole (TMP-SMX) blocks nucleotide and amino acid synthesis in the bacteria. Few case reports have demonstrated the effectiveness of TMP-SMX in the treatment of acne, but data is limited. Similarly, sparse data exist on the use of penicillins and cephalosporins.

Sarecycline is proposed to have a more favorable side effect profile due the narrow-spectrum and preservation of the gut microbiome. In an in vitro study, both doxycycline and minocycline demonstrated a significant decrease in microbial diversity which did not recover after withdrawal of the antibiotics. Sarecycline had a transient decrease in bacterial diversity at the start of treatment but recovered after sarecycline was discontinued [11]. A recent study compared the effect of sarecycline to minocycline against microorganisms commonly found in the gut using in vitro minimum inhibitory concentration (MIC) and time-kill kinetic assays. Overall, sarecycline demonstrated reduced antimicrobial activity against 79% of the tested gut microorganisms [12], further supporting that sarecycline preserves the gut microbiota compared to other tetracyclines. It is possible that differences in gut microbiota differ between nationalities [13], which could potentially have implications on the effects of long-term antibiotic use and may account for the increased efficacy in Hispanics in this post hoc analysis.

Previous generations of tetracyclines have been associated with the development of hyperpigmentation. Studies have shown that hyperpigmentation is independently associated with tetracycline use [14,15]. Historically, minocycline-induced pigmentation has been of concern, with three clinical variants observed. The first is blue-black pigmentation occurring within scars, the second is bluish pigmentation occurring on normal skin, and the third is brown pigmentation in sun-exposed areas [14]. However, a recent cohort study of 186,158 patients found a higher incidence of doxycycline-associated hyperpigmentation in patients of color compared to white patients. In patients taking minocycline, a lower overall risk of hyperpigmentation was observed, but Black, Hispanic, and Asian patients still had an increased risk of hyperpigmentation compared to white patients [16].

In addition, patients might also experience post-inflammatory hyperpigmentation (PIH) as a sequela of acne. PIH is more common in patients with darker skin types and can be difficult to treat, causing an emotional burden for the patients. Recent studies have focused on exploring the pathogenesis of PIH [17,18]. The inflammatory mediators of acne are believed to stimulate abnormal melanin deposition [17]. While treatments are being explored, many therapies pose a risk of damaging adjacent skin. Treating acne before severe inflammatory lesions develop is the best way to prevent development of PIH [18].

As a post hoc analysis, this study faces limitations. The investigators further divided the initial study population, which inevitably increases the likelihood that the observed effects are due to chance. The results of this subgroup analysis warrant confirmation with a randomized controlled trial.

## 5. Conclusions

Sarecycline showed good efficacy and tolerability in phase 3 studies. Doxycycline, another tetracycline antibiotic, is the current first line systemic antibiotic for acne [5]. Doxycycline, however, is limited by adverse effects and the potential for antibiotic resistance. Recently, there has been an increase in focus on skin of color research. Much of the focus is on diagnosis, but it is important to investigate therapies as well. This post hoc analysis aimed to analyze the efficacy of sarecycline in Hispanics and found sarecycline to be an efficacious and well tolerated treatment.

## Data Availability

Not applicable.
